# Toughening Effect of Micro-Cracks on Low-Temperature Crack Propagation in Asphalt Concrete

**DOI:** 10.3390/ma18112429

**Published:** 2025-05-22

**Authors:** Jianhuan Du, Xianxing Dai, Qingyang Liu, Zhu Fu

**Affiliations:** 1 School of Architecture and Civil Engineering, Chengdu University, Chengluo Avenue No. 2025, Chengdu 610106, China; liuqingyang16@stu.cdu.edu.cn (Q.L.); fuzhu@stu.cdu.edu.cn (Z.F.); 2 School of Intelligent Construction, Sichuan Vocational and Technical College, Suining 629000, China; sysznin@163.com

**Keywords:** road engineering, asphalt concrete, micro-cracks, modulus damage, toughening effect, stress field distribution

## Abstract

Asphalt concrete has a unique low-temperature fracture mechanism due to the complex interaction between macro- and micro-cracks. This study investigated the toughening effect of micro-cracks on the crack propagation behavior of asphalt concrete at low temperatures. The Taylor model was applied to establish a modulus damage model of asphalt concrete. In combination with the discrete element method (DEM), a 2D microstructure damage model of asphalt concrete with heterogeneity (aggregate, mortar, and voids) and multi-level (aggregate gradation) characteristics was constructed. A virtual semi-circular bending (SCB) test was performed to reveal the toughening effect of the micro-cracks in terms of macroscopic and microscopic parameters, such as the modulus damage variable, dynamic parameters associated with the main crack propagation, and stress field distribution, laying a foundation for predicting the propagation behavior and path of macroscopic cracks in asphalt concrete. The results showed that (1) the proposed modulus damage model based on the Taylor model produced results that were in good agreement with the numerical simulation (virtual SCB test) results. With an increase in the micro-crack density, the influence of the main cracks on the modulus damage of asphalt concrete gradually reduced, indicating that the micro-cracks exhibited a toughening effect on the main crack propagation; (2) At the meso-scale, the toughening effect of the micro-cracks extended the duration of the crack propagation stage and macro-crack formation stage; that is, the toughening effect of the micro-cracks had a shielding effect on the main crack propagation; (3) The toughening effect could inhibit the shear stress field, contributing to preventing the deterioration in the modulus of asphalt concrete.

## 1. Introduction

Asphalt concrete is a heterogeneous composite material with a specific spatial structure formed by asphalt mortar, aggregates, and voids. During its service period, a large number of micro-cracks can form near the macro-crack tip. Under external loading, these micro-cracks have a shielding effect on macro-crack propagation [1], thereby changing the propagation behavior of macro-cracks. Hence, it is essential to reveal the low-temperature fracture mechanism of asphalt concrete to effectively clarify the influence of micro-cracks on the propagation of macro-cracks with different configuration characteristics, which is also the basis for predicting the propagation and evolution behaviors of cracks in asphalt concrete.

Aiming at the problem of micro-cracks with a unique distribution, the Muskhelishvili complex function [2,3], the method of small parameter [4,5,6], the Gauss–Chebyshev quadrature [7,8], the Brueckner theory [9], and the crack line analysis [10] have been employed to obtain the stress intensity factor (SIF), revealing the toughening effect of micro-cracks with a unique distribution on the main crack propagation. However, in practice, it is difficult to accurately obtain an analytical solution to the SIF under the influence of randomly distributed micro-cracks.

Hence, to reveal the relationship between randomly distributed micro-cracks and the main cracks, Jiang [11,12] and Renshaw [13] established a damage model from the perspective of two equal parallel cracks and two unequal parallel cracks and studied the influence of the interaction between these cracks on the propagation behavior of macro-cracks. In other studies, based on the damage model, scholars have revealed the mechanism of interaction between micro- and macro-cracks from different perspectives, such as closely spaced cracks [14], two collinear cracks and offset cracks [15], parallel micro-cracks [16], and interfacial collinear cracks [17].

Based on the aforementioned results, an interaction model between micro-cracks and main cracks was established, and a series of laboratory tests investigating their interactions was conducted to further refine and optimize the model. Edge-notched disc bend and edge-notched disc compression tests have been performed to study the composite fracture behavior of asphalt concrete under the influence of crack configuration [18,19,20]. Combined with the finite element method (FEM), a semi-circular bending (SCB) test was conducted to reveal the multi-scale cracking behavior by considering the influence of material properties [21], random aggregate distribution [22], and temperature [23]. Moreover, combined with laboratory tests and the FEM, the shielding effect of coplanar cracks on the propagation behavior of macro-cracks was revealed in terms of the micro-crack spacing [24], relative position between cracks [25], crack size [26,27], and crack depth [28]. The Kalthoff–Winkler experiment was performed to reveal the toughening mechanism of micro-cracks with a unidirectional distribution on the propagation of main cracks by considering the number of micro-cracks [29]. With the application of the acoustic emission technology and the digital image correlation in laboratory tests, the transient propagation behavior of main cracks under the influence of micro-cracks has also been revealed [30,31].

Since the practical problems of crack interaction are usually three-dimensional (3D), two-dimensional (2D) cracks often have difficulty reflecting the propagation behavior of external cracks and their influence on the propagation of internal cracks. However, it is complex and difficult to establish the theoretical model and numerical model of the crack propagation by considering the influence of the cracks’ spatial distribution and degree of freedom in three dimensions (3D). Therefore, most investigations on crack propagation are still limited to two-dimensional (2D) crack arrays.

In summary, most studies have focused on theoretically revealing the interaction between micro-cracks and main cracks by establishing a damage model. Combined with laboratory tests and the FEM, the material configuration, temperature, and micro-crack configuration characteristics have been typically considered to study the shielding effect of micro-cracks on the propagation behavior of main cracks. However, in practice, micro-cracks with a random distribution significantly change the stress field distribution at the main crack tip, thereby changing the propagation behavior of the main cracks and the macro-fracture behavior of asphalt concrete.

Hence, based on previous results, asphalt concrete with an AC-13 aggregate gradation (suspension-dense structure) was taken as the research subject, and the Taylor model and DEM were applied to establish a heterogeneous, multi-level, 2D micro-structural damage model of this asphalt concrete by introducing the micro-crack density as a parameter describing the distribution and number of micro-cracks. A virtual SCB test was conducted to reveal the toughening effect of micro-cracks on the propagation behavior of the main crack from the modulus damage, dynamic parameters of the main crack propagation, and the stress field distribution. This study aimed to clarify the toughening effect of micro-cracks and provide a theoretical basis for predicting the macro-crack behavior and crack propagation path of asphalt concrete.

## 2. Modulus Damage Model of Asphalt Concrete

### 2.1. Modulus Damage Model Based on Main Crack Configuration Characteristics

**Figure 1:**
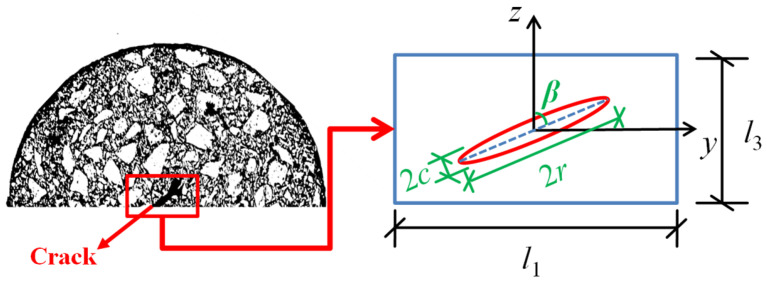
Schematic of the representative volume element (RVE) of a thin elliptical main crack.

For asphalt concrete, the representative volume element (RVE) can be selected to simulate the main crack, as shown in Figure 1. In the figure, *l*_1_ and *l*_3_ denote the length and height of the RVE, respectively. Based on Eshelby’s equivalent inclusion theory [32], the main crack is considered a thin ellipse, and its configuration characteristics are represented by the crack deflection angle *β* (the angle between the crack propagating direction and loading direction) and crack size (crack thickness 2*c*, crack length 2*r* in the other two directions).

Based on Mori-Tanaka’s theory [33], the modulus damage variable *D* due to the main crack can be expressed as follows:

(1) $$\;D=1-\frac{E_{T}}{E}=1-\frac{1}{1+\frac{f_{1}}{f_{0}}(\frac{4(1-v)r}{\pi c})}$$

Here, *E* denotes the modulus of the asphalt concrete without the main crack; *E*_T_ denotes the effective modulus of the asphalt concrete with the main crack; *f*_0_ and *f*_1_ denote the volume fractions of the asphalt concrete and the main crack, respectively, and *f*_0_ *=* 1 *− f*_1_; *ν* represents the Poisson’s ratio.

Combined with the RVE (as shown in Figure 1), the volume fraction of the main crack can be expressed by the size of the RVE and the main crack configurations, i.e.,

(2) f1f0=4πr2c3l12l3rc=43π0p32tan⁡β

Here, *α* represents the shape ratio of the main crack, i.e., *α* = *c*/*r*; *p* represents the area fraction (the ratio of the main crack area to the bottom area of the RVE, i.e., *p* = π*r*^2^/$l_{1}^{2}$); *β* represents the crack deflection angle (0° < *β* < 90°), and tan*β* = *l*_1_/*l*_3_ (it is related to the main crack distribution).

The modulus damage model of asphalt concrete related to the main crack configuration characteristics can be obtained by combining Equations (1) and (2):

(3)  D=1−ETE=1−11+16(1−v)tanβ3f0(pπ)32

**Figure 2:**
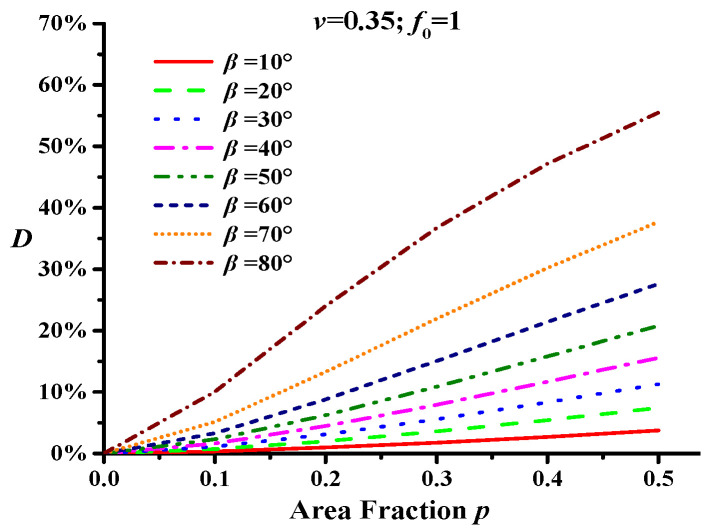
Modulus damage curve of asphalt concrete with a main crack.

The Poisson ratio *v* of asphalt concrete is generally between 0.25 and 0.45, and most studies have assumed a Poisson ratio of 0.35 [34,35]. Considering that *v* decreases at low temperatures, it was set to 0.3 in this study. Moreover, because there is one main crack in asphalt concrete, its volume fraction can be considered negligible (*f*_1_ ≈ 0), i.e., *f*_0_ ≈ 1. According to Equation (3), the relationship between the modulus damage variable *D* of asphalt concrete and the configuration characteristics of the main crack can be obtained, as shown in Figure 2 (*v* = 0.35; *f*_0_ = 1).

Figure 2 shows that the changes in the main crack configuration result in an increase in the modulus damage variable *D*, implying a reduction in the effective modulus of asphalt concrete. Under the same crack deflection angle *β*, the modulus damage variable *D* gradually increases with increasing crack length (i.e., the area fraction *p* increases), and the increasing trend becomes evident when *β* exceeds 30°. For the same crack length, *D* evidently increases with an increase in *β*, and the crack deflection angle has a more evident influence on the modulus damage variable than the crack length. The results shown in Figure 2 indicate that the crack deflection angle *β* significantly influences the modulus damage of the asphalt concrete.

### 2.2. Toughening Effect of Micro-Cracks

The random distribution of micro-cracks in asphalt concrete changes the damage behavior of the main crack, thereby changing the fracture mode. Therefore, a simple and effective method is presented based on the concept of the effective field to analyze the interaction of micro-cracks of a large number or of a high density, i.e., the Taylor model [36]. And, in order to establish a relationship between the micro-cracks and the modulus damage behavior, the interaction of micro-cracks in a circular or elliptical region was considered by the Taylor model, while the influence of all other micro-cracks is reflected by modifying the stress applied in the far field.

According to the Taylor model, the following assumptions are typically made in the damage modeling of asphalt concrete with micro-cracks [37]:

1. Micro-crack closure and crack surface friction are neglected.
2. The micro-crack distribution is random.
3. Only weak interactions exist between neighboring micro-cracks.

Based on these assumptions and combined with Equation (3), the relationship between the micro-cracks and the modulus damage can be expressed as follows:

(4) $$D^{\prime }=1-\frac{E^{\prime }}{E_{T}}=1-{\left[1+f_{2}\frac{16(1-v^{2})(10-3v)}{45(2-v)}\frac{E}{E_{T}}\right]}^{-1}$$

Here, *D*′ denotes the modulus damage variable under the toughening effect of micro-cracks; *E*′ denotes the effective modulus of asphalt concrete with micro-cracks and a main crack; and *f*_2_ denotes the micro-crack density, which is related to the number, tendency, and geometry configuration of the micro-cracks [38].

(5) $$\left\{\begin{array}{c} f_{2}=\frac{4}{3}\pi cr^{2}\left(\frac{N}{\Omega }\right)\;\;\;\;\;\;\;\;\;\;\;\;\;\;\;\;\;\;\;\;\;3D\\ f_{2}=\left(\frac{N}{\Omega }\right)\;\;\;\;\;\;\;\;\;\;\;\;\;\;\;\;\;\;\;\;\;\;\;\;\;\;\;\;\;\;\;\;\;2D \end{array}\right.$$

Here, *N* denotes the number of micro-cracks, and Ω denotes the total volume of asphalt concrete (3D) and the total area of asphalt concrete (2D).

**Figure 3:**
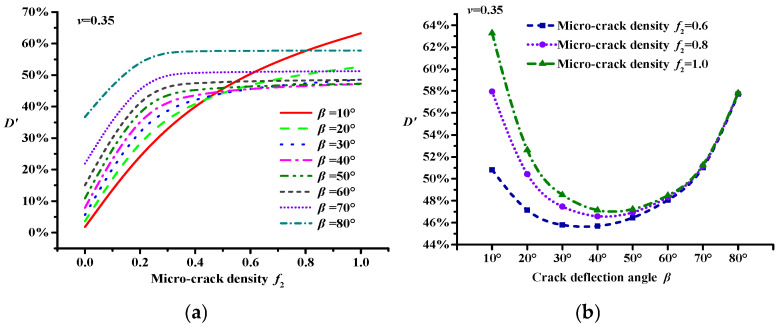
Modulus damage model considering the toughening effect of micro-cracks. (**a**) Modulus damage curve with different micro-crack density. (**b**) Modulus damage curve under high micro-crack densities.

In this study, the Poisson ratio *v* was set to 0.3, and the area fraction *p* was 0.3. Different crack deflection angles (*β*) were chosen: 10°, 20°, 30°, 40°, 50°, 60°, 70°, and 80°. The micro-crack densities were 0.0, 0.2, 0.4, 0.6, 0.8, and 1.0 [39]. According to the result from Figure 2, the relationship curve between the micro-cracks and the modulus damage can be obtained using Equation (4), as shown in Figure 3 (*v* = 0.35).

When *β* = 10°, the modulus damage variable *D*′ increases significantly with the increase in the micro-crack density (see Figure 3a). This is because the angle between the crack deflection and the direction of the load is small, resulting in a small interaction between the macro-crack and the micro-crack. This makes it difficult to produce the crack toughening effect.

The results shown in Figure 3a indicate that when *β* is above 10°, the change in the modulus damage variable *D*′ gradually stabilizes with increasing micro-crack density. When the micro-crack density is below 0.6 (*f*_2_ < 0.6), with an increase in *β*, the modulus damage variable *D*′ also increases; otherwise, under a high micro-crack density (1.0 ≥ *f*_2_ ≥ 0.6), *D*′ first decreases and then increases (see Figure 3b). This indicates that micro-cracks with a low density lead to an expansion of the damage zone at the main crack tip, which aggravates the modulus damage of the asphalt concrete, whereas high-density micro-cracks inhibit this expansion, thereby reducing the modulus damage of the asphalt concrete, i.e., the micro-cracks exhibit a toughening effect.

Moreover, according to Equations (3) and (4), the modulus damage variable *D*′ is related to the volume fractions of the asphalt concrete and the macro-crack. For asphalt concrete of different gradations with one macro-crack, the results have certain similarities. However, as the number of macro-cracks increases, the micro-crack density threshold effect at crack toughening gradually decreases.

Based on the aforementioned results, the DEM was applied to establish a 2D mesoscopic damage model of the asphalt concrete. A virtual SCB test was conducted to study the modulus damage, dynamic response of main crack propagation, and stress field distribution in the crack tip region and thus elaborate the toughening effect of micro-cracks.

## 3. Mesoscopic Damage Model of Asphalt Concrete

### 3.1. Meso-Parameter of Asphalt Concrete

Based on laboratory test results [40], a polyphosphoric acid (PPA)–styrene butadiene styrene (SBS) composite modified asphalt was adopted, with the PPA and SBS contents being 1% and 3%, respectively. The performance grade (PG) was PG 70-28. Table 1 and Table 2 present the gradation and physical indicators of the studied asphalt concrete (AC-13 suspension-dense structure). The source of the specimens is the author’s lab.

**Figure 4:**
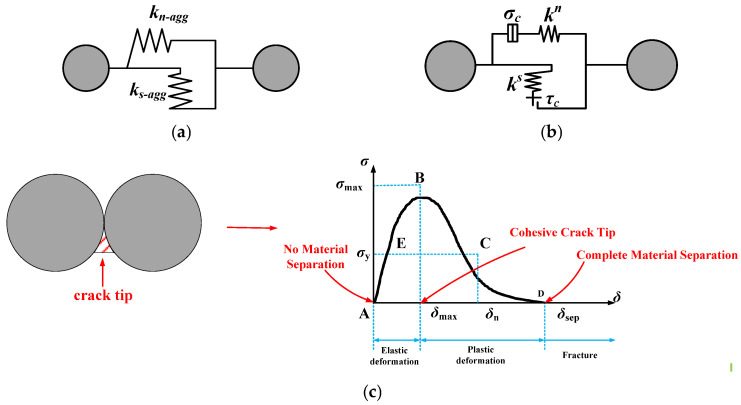
Mechanical contact models for asphalt concrete. (**a**) Linear model between aggregate particles. (**b**) Parallel-bond model between asphalt mortar particles. (**c**) Cohesive zone model between aggregate and asphalt mortar particles.

Asphalt concrete is a typical heterogeneous composite material. Therefore, aggregate particles with irregular shapes can be established using the random aggregate generation algorithm, and the voids and asphalt mortar can be constructed by controlling the void ratio. There are three contact behaviors in the DEM model of asphalt concrete: contact between coarse aggregate particles, contact between asphalt mortar particles, and contact between coarse aggregate particles and asphalt mortar particles. These contact behaviors can be defined by different contact models [41,42], as shown in Figure 4.

The linear model can describe the contact between aggregate particles which are purely elastic (see Figure 4a), i.e.,

(6) $$\left\{\begin{array}{c} k_{n-agg}=4E_{s}R\\ k_{s-agg}=\frac{2E_{s}R}{1+v_{s}} \end{array}\right.$$

Here, *k_n-agg_* and *k_s-agg_* denote the normal and tangential stiffnesses of the aggregates; *E_s_* and *ν_s_* denote the dynamic modulus and Poisson’s ratio of the aggregates, respectively. *R* represents the radius of an aggregate particle (it is generally taken as 1 mm). From the results of laboratory tests on basaltic crushed rock [43,44], the dynamic modulus, Poisson’s ratio, tensile strength, and coefficient of internal friction were taken as 55.5 GPa, 0.25, 27.6 MPa, and 0.5, respectively.

The parallel bond model can describe the contact between asphalt mortar particles (see Figure 4b), and the meso-parameters can be expressed as follows:

(7) $$\left\{\begin{array}{c} k^{n}=\frac{E_{a}}{\sqrt{3}(1+v_{a})(1-2v_{a})}\\ k^{s}=\frac{E_{a}(1-4v_{a})}{\sqrt{3}(1+v_{a})(1-2v_{a})} \end{array}\right.$$

Here, *k^n^* and *k^s^* denote the internal normal and tangential stiffnesses of the asphalt mortar, and *E*_a_ and *ν*_a_ denote the dynamic modulus and Poisson’s ratio of the asphalt mortar, respectively.

The cohesive zone model (CZM) describes the contact between aggregate particles and asphalt mortar particles, as shown in Figure 4c. The contact force *σ* between asphalt mortar particles and aggregate particles is calculated via a stress measuring circle. The stress measuring circle records the particle position, rotation angle, and contact force between particles, i.e.,

(8) $$\;\sigma =\sqrt{{\left(\sigma_{c}^{n}\right)}^{2}+{\left(\tau_{c}^{n}\right)}^{2}}$$

Here, $\sigma_{c}^{n}$ and $\tau_{c}^{n}$ denote the normal and tangential contact forces, respectively.

The maximum contact force *σ*_max_ can be obtained from the normal force *σ*_c_, tangential force *τ*_c_, and angle *φ*:

(9) $$\sigma_{max}=\left(1-\frac{2\varphi }{\pi }\right)\times \sigma_{c}+\frac{2\varphi }{\pi }\times \tau_{c}$$

Here, *φ* represents the angle between the direction of the contact force and the connecting line to the particle center.

When the contact force *σ* is greater than the maximum contact force *σ*_max_ (*σ* > *σ*_max_), the contact begins to yield or soften, indicating a fracture.

Table 3 presents the meso-parameters of the asphalt concrete at a temperature of −20 °C [1,40,45].

### 3.2. Construction of Micro-Cracks and Main Crack

Through the particle flow code (PFC2D), an SCB model of the asphalt concrete, which is a semi-circle specimen with a radius of 75 mm, was established using the random aggregate generation algorithm. Moreover, the commands “dfn addracture” and “dfn Generate” can be used to define the main crack with different configurations and micro-cracks with a uniform orientation and random distribution.

The research results [1] showed that micro-cracks with a high micro-crack density (1.0 ≥ *f*_2_ ≥ 0.6) exhibit a crack shielding effect, which delays the propagation of the main crack with the specific configuration (crack deflection angle *β* = 45°). Therefore, to further illustrate the relationship between the toughening effect of micro-cracks and the main cracks with different configurations, based on the aforementioned research results (see Section 2.2), the micro-crack density (*f*_2_) values were taken as 0.0 and 0.6, respectively; and the main crack deflection angle (*β*) values were taken as 0°, 22.5°, 45°, and 67.5°, respectively; and the main crack length was taken as 2 mm. The crack length of the micro-cracks was set to 1 mm, consistent with the Taylor model’s assumption that only weak interactions exist between neighboring micro-cracks.

And the generation tendency angle of micro-cracks is 0° to 360°, and the generation range is within the SCB model. It is controlled by calculating the ratio of the number of micro-cracks inside the model to the total area of the model.

**Figure 5:**
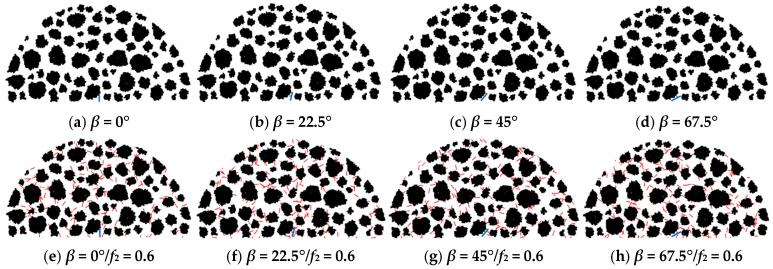
Meso-damage model of asphalt concrete based on the DEM.

Figure 5 shows the meso-damage model of the asphalt concrete, where the black particles represent aggregates, the blue line represents the main crack, and the red line represents micro-cracks with a random distribution.

### 3.3. Virtual SCB Test

**Figure 6:**
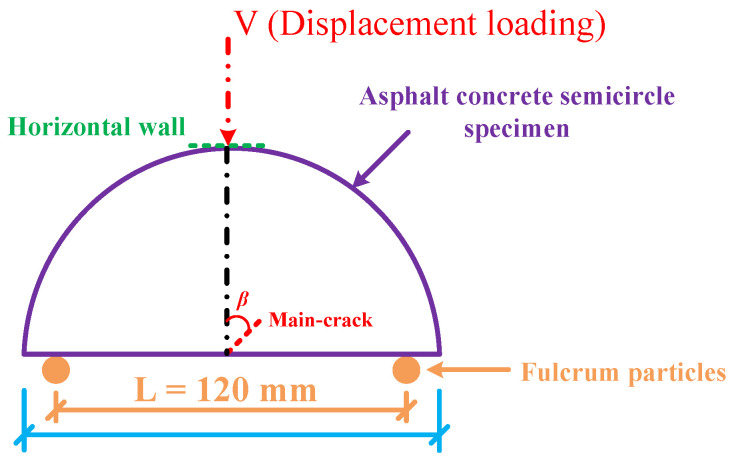
Schematic of the virtual SCB test.

The virtual SCB test was conducted through the PFC2D, as shown in Figure 6. Two immovable rigid spherical particles were set at the lower part of the specimen as fulcrums, separated by a distance of 120 mm; a horizontal wall was set on the upper part of the specimen to simulate the indenter in the universal testing machine (UTM) test system (the length of the horizontal wall was the same as that of the indenter). In addition, a constant displacement loading of 1 mm/min was applied to prevent the rapid propagation of the main crack caused by the excessive loading rate.

## 4. Crack Propagation Characteristics Under the Toughening Effect of Micro-Cracks

### 4.1. Modulus Damage of Asphalt Concrete

**Figure 7:**
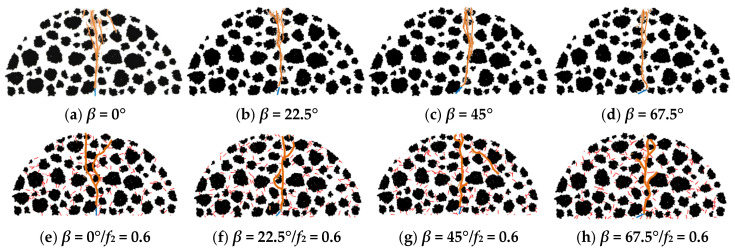
Macro-crack propagation in asphalt concrete.

Previous numerical simulation and experimental results [46] indicate that the numerical simulation is accurate. In PFC2D, the generation of cracks mainly depends on the breakage of the force chain at the particle contact interface. If the force chain between particles undergoes normal fracture, an I-mode crack is produced. If the force chain between particles undergoes tangential fracture, a II-mode crack is produced. Figure 7 shows the virtual SCB test results, where the orange line represents the macro-crack.

**Figure 8:**
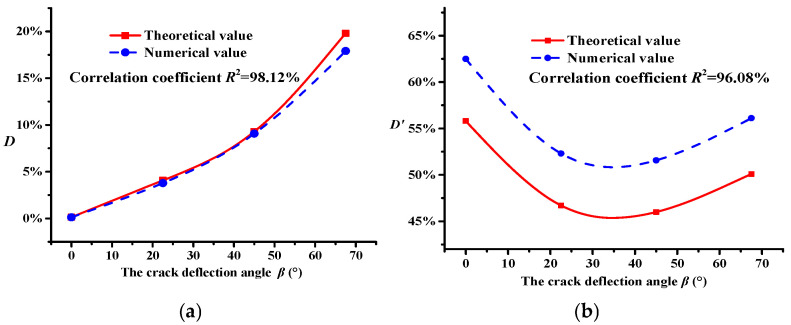
Modulus damage variable curve of asphalt concrete. (**a**) Modulus damage variable *D*. (**b**) Modulus damage variable *D*′.

Clearly, compared with the crack propagation without the toughening effect of micro-cracks, there are significant differences in the macro-crack distribution under the influence of micro-cracks, particularly in terms of the branch cracks and the deflection of the macro-crack (see Figure 7). Moreover, the curve of the modulus damage variable with different main crack configurations can be obtained by extracting the numerical simulation results, as shown in Figure 8.

Figure 8 shows that there is a difference between the theoretical and numerical results, because the skeleton structure of the material has an impact on the crack propagation and evolution, but the theoretical model assumes ideal conditions and simplifications that only consider the modulus of the material with a different skeleton structure, ignoring the influence of skeleton structure characteristics on crack propagation. The numerical methods often involve the influence of the aggregate skeleton characteristics. However, there is a similarity between the modulus damage curves obtained using the Taylor model and the DEM, respectively.

Moreover, combined with the results shown in Figure 7, under the toughening effect of micro-cracks, although the changes in the crack propagation path are similar with different main crack configurations, the modulus damage variable *D* significantly increases, implying that the main crack propagated easily. Otherwise, under the toughening effect of micro-cracks, the modulus damage variable *D*′ first decreased and then increased with increasing crack deflection angle *β*. The modulus damage variable *D*′ was minimum when *β* was approximately 30° (see Figure 8b), indicating that the micro-cracks had a strong shielding effect on the main crack propagation, making it difficult for the main crack to propagate. This result confirms the evident toughening effect provided by the micro-cracks.

The toughening effect of micro-cracks on the modulus damage of asphalt concrete was studied from a macroscopic perspective. To further illustrate the influence of micro-cracks on the main crack propagation from a mesoscopic perspective, the dynamic parameters associated with the main crack propagation process were extracted as follows.

### 4.2. Dynamic Parameters in the Main Crack Propagation Process

Through the PFC2D discrete element analysis platform, the dynamic parameters of the crack propagation can be obtained using the crack propagation monitoring program. Based on the DEM, the number of particle contact failures represents the number of cracks, i.e., a higher number of particle contact failures implies a greater number of cracks, indicating that more energy is required for crack propagation. In addition, an increment in the logarithmic time step describes the duration of the three fracture stages [46], namely the crack incubation stage, the crack propagation stage, and the macro-crack formation stage. Table 4 presents the results of the dynamic parameters related to crack propagation.

In Table 4, it can be seen that without the influence of micro-cracks, an increase in the crack deflection angle *β* gradually reduces the duration of the three fracture stages; moreover, there is a gradual reduction in the number of cracks in the crack propagation stage and macro-crack formation stage. This result implies that an increase in *β* easily promotes crack propagation, thereby accelerating the damage process of asphalt concrete. In terms of the macro-performance, the modulus damage in asphalt concrete is aggravated (see Figure 8a).

Compared with the former results mentioned above, the micro-cracks in the asphalt concrete further reduced the duration of the crack incubation stage (see Table 4), resulting in further aggregation of modulus damage in asphalt concrete (see Figure 8b). However, the duration of the crack propagation stage and the macro-crack formation stage significantly increased (see Table 4), indicating that the micro-cracks exhibited a crack toughening effect on the main crack propagation, i.e., it was difficult for the main crack to propagate. These results show that micro-cracks with a micro-crack density *f*_2_ = 0.6 were beneficial to delaying the modulus damage of asphalt concrete (see Figure 3), indicating that the micro-cracks had a toughening effect.

Under the toughening effect of micro-cracks, with an increase in the main crack deflection angle *β*, the duration of the crack incubation stage showed a trend of first increasing and then decreasing (see Table 4), indicating that the micro-cracks reduced the modulus damage of the asphalt concrete given that they could effectively delay the crack occurrence, as shown in Figure 8b. Moreover, from Table 4, it can be seen that with an increase in *β*, the number of cracks and the duration of the crack propagation stage and macro-crack formation stage show a trend of first increasing and then decreasing, implying that the crack toughening effect of micro-cracks occurred in these two stages. These results indicate that the toughening effect of micro-cracks changed the main crack propagation behavior, resulting in differences in the macro-crack distribution, as shown in Figure 7.

Hence, it is necessary to illustrate the toughening effect of micro-cracks by studying the stress field distribution.

### 4.3. Stress Field Distribution

**Figure 9:**
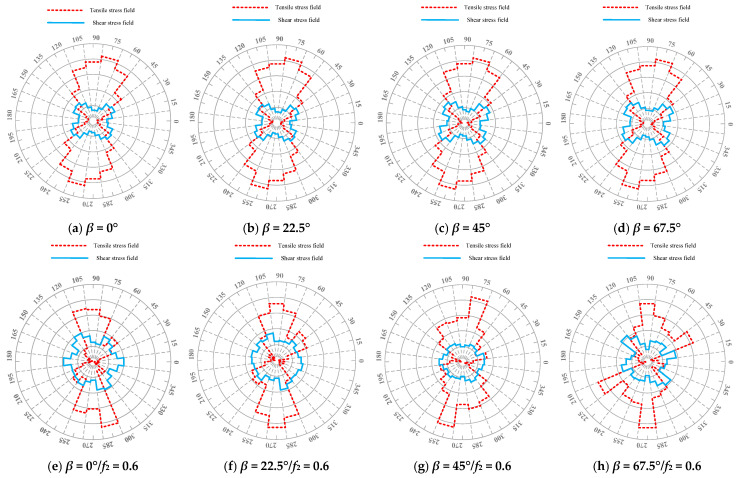
Stress field distribution in asphalt concrete.

**Figure 10:**
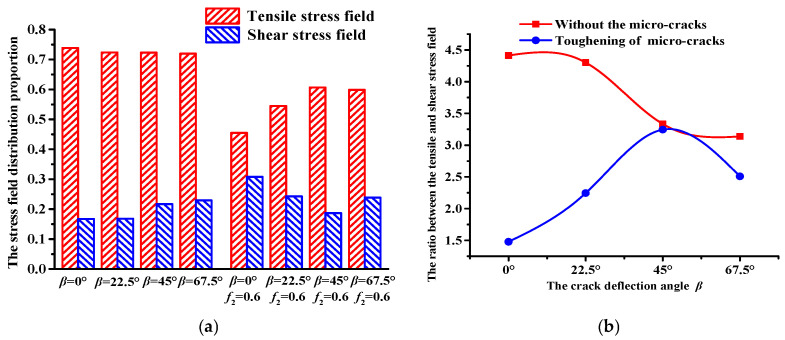
Proportion of the stress field distribution in asphalt concrete. (**a**) The tensile and shear stress field distribution proportion. (**b**) Ratio of the tensile stress field to the shear stress field.

A subprogram was programmed using the FISH language to identify the stress field distribution, as shown in Figure 9, where the tensile stress field distribution and shear stress field distribution are described by red and blue lines, respectively. Moreover, the distribution proportion of the stress field can be obtained from Figure 9, as shown in Figure 10.

Figure 9 shows that as the crack deflection angle *β* increases, there are no significant changes in the stress field distribution in asphalt concrete without the influence of the micro-cracks. An increase in *β* causes a decrease in the tensile stress field distribution and an increase in the shear stress field, resulting in a decrease in the ratio between the tensile and shear stress fields (see Figure 10). This shows that without the influence of the micro-cracks, the fracture mode of the asphalt concrete gradually changes from mode-I fracture (mainly tensile fracture) to mode-II fracture (mainly shear fracture).

On the other hand, under the toughening effect of micro-cracks, the stress field distribution of the asphalt concrete changes significantly (see Figure 9). Moreover, compared with the results of the asphalt concrete without the influence of micro-cracks, Figure 10 shows that the proportion of the tensile stress field distribution is lower than that of the shear stress field distribution.

With an increase in the crack deflection angle *β*, under the toughening effect of micro-cracks, the proportion of the tensile stress field distribution gradually increases while the proportion of the shear stress field distribution gradually decreases, contributing to the first increasing and subsequently decreasing trend in the ratio between the tensile and shear stress fields (see Figure 10). These results indicate that the toughening effect of micro-cracks can inhibit the generation of mode-II cracks, contributing to the delay in the time experienced in the crack propagation stage and macro-crack formation stage (see Table 4) and reducing the modulus damage of asphalt concrete (see Figure 8). This means that the toughening effect of micro-cracks brings about an improvement in the fracture toughness of asphalt concrete.

## 5. Conclusions

In this study, the Taylor model was applied to establish a modulus damage model of asphalt concrete, and combined with the DEM, a modulus damage model of the concrete with a meso-structure was constructed using the random aggregate generation algorithm. Moreover, a virtual SCB test was performed to illustrate the toughening effect of micro-cracks in terms of the modulus damage, dynamic parameters related to crack propagation, and stress field distribution. The conclusions drawn from the study are as follows:

(1) The damage modulus results obtained from the Taylor model and numerical simulation showed a good correlation. Under the toughening effect of micro-cracks, the modulus damage variable D′ first decreased and then increased with increasing crack deflection angle β, indicating a reduction in the modulus damage of the asphalt concrete, i.e., an improvement in its fracture toughness.

(2) In terms of the dynamic parameters related to crack propagation, due to the toughening effect of micro-cracks, the durations of the crack propagation and macro-crack formation stages were prolonged, implying that the micro-cracks showed a crack shielding effect on the main crack propagation.

(3) The stress field distribution varied significantly because of the toughening effect of micro-cracks. The ratio between the tensile and shear stress field distributions first increased and then decreased, implying that the fracture mode of the asphalt concrete gradually changed from mode-II fracture to mode-I fracture, i.e., the toughening effect of micro-cracks could inhibit the appearance of mode-II cracks in the concrete.

Our findings provide a theoretical basis and technical support for illustrating the propagation behavior and propagation path of macro-cracks in asphalt concrete and lay a foundation for revealing its crack-resistance mechanism.

The two-dimensional model encounters difficulties in capturing the interaction between out-of-plane and in-plane crack propagation, and its capacity to represent three-dimensional directional crack growth behavior is constrained. To overcome these limitations in two-dimensional spatial analysis, future research should focus on integrating the spatial configuration of the cracked body, as well as the heterogeneous spatial distribution of aggregates and voids. These elements are essential for developing a more precise and holistic understanding of crack propagation mechanics within three-dimensional domains.

## Figures and Tables

**Table 1:** Gradation of AC-13 asphalt concrete.

Aperture size (mm)	16.0	13.2	9.5	4.75	2.36	1.18	0.60	0.30	0.15	0.075
Passing ratio (%)	100.0	97.5	84.0	62.5	42.5	32.0	24.0	15.5	11.0	6.0

**Table 2:** Physical indicators of AC-13 asphalt concrete.

Oil–Stone Ratio (%)	Gross Volumetric Density (g/cm^3^)	Marshall Stability (kN)	Porosity (%)	Flow Value (mm)	Void Filled with Asphalt (VFA) (%)	Maximum Theoretical Density (g/cm^3^)
6.27	2.547	12.48	2.1	4.34	76.2	2.610

**Table 3:** Meso-parameters of asphalt concrete at a temperature of −20 °C.

Particle Contact	Meso-Parameters	
Aggregate	Dynamic modulus (GPa)	55.5
	Tensile strength *σ* (MPa)	27.6
	Poisson’s ratio *ν_s_*	0.23
	Normal stiffness *k_n_* (MPa)	222
	Tangential stiffness *k_s_* (MPa)	90.24
Asphalt concrete	Void ratio (%)	2.1
	Particle density (kg∙m^−3^)	2582
Asphalt mortar	Interparticle contact modulus *E_c_* (GPa)	0.832
	Particle normal to the tangential stiffness ratio *k^n^*/*k^s^*	1
	Parallel bond modulus $E_{c}^{\prime }$ (GPa)	0.596
	Parallel bond normal to tangential stiffness ratio *k^n^_c_*/*k^s^_c_*	0.667/0.133
	Interparticle friction coefficient *f_s_*	0.5
	Average normal strength of parallel bond *σ*_c_ (MPa)	3.553
	Standard deviation of parallel bond normal strength (MPa)	1
	Average tangential strength of parallel bond *τ*_c_ (MPa)	3.553
	Standard deviation of the parallel bond tangential strength (MPa)	1

**Table 4:** Dynamic parameters related to crack propagation.

Micro-Crack Density *f*_2_	Crack Deflection Angle *β*	Crack Incubation Stage		Crack Propagation Stage		Macro-Crack Formation Stage	
		Crack Number (Items)	∆_1_	Crack Number (Items)	∆_2_	Crack Number (Items)	∆_3_
0.0	0°	1	4.401	36	0.253	593	0.030
	22.5°	1	4.398	33	0.240	462	0.023
	45°	1	4.395	26	0.215	446	0.020
	67.5°	1	4.380	14	0.145	388	0.020
0.6	0°	1	4.346	11	0.306	131	0.033
	22.5°	1	4.285	12	0.356	137	0.037
	45°	1	4.210	14	0.700	144	0.040
	67.5°	1	4.110	13	0.546	135	0.032

(Note: ∆_1_, ∆_2_, and ∆_3_ denote the durations of the crack incubation stage, crack propagation stage, and macro-crack formation stage, respectively).

## Data Availability

The original contributions presented in this study are included in the article. Further inquiries can be directed to the corresponding author.

## Nomenclature

DEM	Discrete Element Method
SCB	Semi-Circular Bending
FEM	Finite Element Method
RVE	Representative Volume Element
*β*	Crack deflection angle
2*c*	Crack thickness
2*r*	Crack length
*D*	Modulus damage variable
*E*	Modulus of the asphalt concrete without the main crack
*E*_T_	Effective modulus of the asphalt concrete with the main crack
*f*_0_	Volume fractions of the asphalt concrete
*f*_1_	Volume fractions of the main crack
*ν*	Poisson ratio
*α*	Shape ratio of the main crack
*p*	Area fraction
*D*′	Modulus damage variable under the toughening effect of the micro-cracks
*E*′	Effective modulus of asphalt concrete with micro-cracks and a main crack
*f*_2_	Micro-crack density
PPA	Polyphosphoric acid
SBS	Styrene butadiene styrene
PG	Performance grade
*K_n-agg_*	Normal stiffness of the aggregates
*K_s-agg_*	Tangential stiffness of the aggregates
*E_s_*	Dynamic modulus of the aggregates
*ν_s_*	Poisson ratio of the aggregates
*R*	Radius of an aggregate particle
*k* ^n^	Internal normal stiffnesses of the asphalt mortar
*k* ^s^	Tangential stiffnesses of the asphalt mortar
*E*_a_	Dynamic modulus of the asphalt mortar
*ν*_a_	Poisson ratio of the asphalt mortar
$\sigma_{c}^{n}$	Normal contact forces
$\tau_{c}^{n}$	Tangential contact forces
*σ*_max_	Maximum contact force
*φ*	Angle between the direction of the contact force and the connecting line to the particle center.
PFC2D	Particle flow code
